# Colorectal cancer screening of high-risk populations: A national survey of physicians

**DOI:** 10.1186/1756-0500-5-64

**Published:** 2012-01-24

**Authors:** Pascale M White, Malini Sahu, Michael A Poles, Fritz Francois

**Affiliations:** 1 Division of Gastroenterology and Department of Medicine, New York University Langone Medical Center, New York, NY, USA; 2 Division of Gastroenterology and Department of Medicine, Temple University School of Medicine, Philadelphia, PA, USA

## Abstract

**Background:**

The incidence of colorectal cancer can be decreased by appropriate use of screening modalities. Patients with a family history of colon cancer and of African-American ethnicity are known to be at higher risk of developing colorectal cancer. We aimed to determine if there is a lack of physician knowledge for colorectal cancer screening guidelines based on family history and ethnicity. Between February and April 2009 an anonymous web-based survey was administered to a random sample selected from a national list of 25,000 internists, family physicians and gastroenterologists. A stratified sampling strategy was used to include practitioners from states with high as well as low CRC incidence. All data analyses were performed following data collection in 2009.

**Results:**

The average knowledge score was 37 ± 18% among the 512 respondents. Gastroenterologists averaged higher scores compared to internists, and family physicians, *p *= 0.001. Only 28% of physicians correctly identified the screening initiation point for African-Americans while only 12% of physicians correctly identified the screening initiation point and interval for a patient with a family history of CRC. The most commonly cited barriers to referring high-risk patients for CRC screening were "patient refusal" and "lack of insurance reimbursement."

**Conclusions:**

There is a lack of knowledge amongst physicians of the screening guidelines for high-risk populations, based on family history and ethnicity. Educational programs to improve physician knowledge and to reduce perceived barriers to CRC screening are warranted to address health disparities in colorectal cancer.

## Background

As the third leading cause of malignancy-related death in the United States, colorectal cancer (CRC) is expected to be responsible for over 50,000 deaths in 2011 [1,2]. While various CRC screening efforts have been implemented [3], notable disparities in screening prevalence exist among minorities, those with low incomes, lower education, as well as among individuals without health insurance [3].

While some of the barriers that influence CRC screening rates include patient factors, as delineated above [4-6], there are also physician-related factors that should be considered, such as failure to recommend screening to patients [7-9]. The decision whether or not to adopt a screening strategy might be driven by both physician-perceived as well as real barriers such as patient co-morbidities, prior patient refusal of screening and lack of patient compliance, physician forgetfulness, time restrictions, and a lack of reminder systems and test tracking systems [10,11]. In addition, physician knowledge of current CRC screening guidelines may be an important contributing factor to screening referral practices.

Primary care physician recommendations and screening practices are known to be inconsistent with established national guidelines [12,13]. Given that opportunities for screening referral exist across medical disciplines, investigating physician knowledge about CRC screening guidelines in various specialties might reveal modifiable factors that impact the adoption of screening strategies at physician-patient contact points. Furthermore, high-risk individuals such as those with a family history of colorectal cancer may not be screened as necessary at the appropriate initiation point or interval if not identified appropriately or if the guidelines are unknown [14]. To date, few studies have assessed physician knowledge about colorectal cancer screening guidelines for high-risk patient populations across specialties, nor have any examined physician barriers to appropriate colorectal cancer screening of high-risk patients with a family history of colorectal lesions, or of ethnically diverse patients. We hypothesized that there is a gap in physician knowledge regarding colorectal cancer screening of these high-risk patient populations, and that there are modifiable physician barriers to appropriate colon cancer screening of high-risk patient populations.

We aimed to investigate physician knowledge of CRC screening guidelines based on family history as well as ethnicity among three medical specialties, while also evaluating barriers for compliance with established CRC screening guidelines.

## Methods

### Study population

The accessible population for this study consisted of physician members of the American Medical Association (AMA) who had supplied an email address as part of the registration process. The AMA is a national professional organization whose membership of 240,000 reflects 23% of all US physicians [15].

### Study design

Between February and April 2009 a cross-sectional survey of US physicians was performed using the AMA masterfile. A stratified sampling strategy was used to randomly select physicians from low and high CRC incidence states. States with 34.3-42.0 CRC cases per 100,000 were defined as low incidence, those with 42.1-48.9 were considered moderate, while those with 49.0-59.6 cases were defined as high incidence [16]. All individuals received an email with a cover letter inviting participation in the survey by following a link that registered a unique masked code for each responder but which did not allow for further identification of the participant. A web-based interface was used to allow participants to provide anonymous responses. All answers were compared and scored based on the US multi-society task force and American College of Gastroenterology (ACG) guidelines [17,18]. Survey completion was voluntary and no incentive was provided. Additional written consent was not obtained from survey participants. The Institutional Review Board approved the study protocol.

### Study instrument

A 19-item survey was developed to assess knowledge and adherence barriers to screening guidelines for high-risk and ethnically diverse populations (Additional File 1: Appendix 1). The instrument was tested for face and content validity within our institution among primary care physicians as well as gastroenterologists. The scenarios presented included:

• A patient's father had adenomatous polyps at age 55. At what age would you recommend screening that patient for colorectal cancer and if the exam is normal how often would you screen?

• At what age would you recommend starting to screen your Asian-American patients with no family history of colorectal cancer?

• A patient has a brother and father with colorectal cancer both diagnosed in their 70s. At what age would you recommend screening that patient and if the exam is normal how often would you screen?

• At what age would you recommend starting to screen your African-American patients with no family history of colorectal cancer?

• A patient was told that he has a family history of Familial Adenomatous Polyposis (FAP), but has not been genetically tested. At what age would you recommend screening and if the exam is normal how often would you screen?

### Study outcomes

The primary outcome of this study was physician knowledge of and adherence to the US multi-society task force and ACG guidelines regarding colorectal cancer screening in high-risk populations (Additional File 2: Appendix 2). A score was calculated as a percentage of correct responses for questions assessing knowledge. The secondary aim was to determine physician barriers to appropriate screening practices.

### Statistical analysis

Descriptive statistics were performed on all variables assessed by our instrument. The frequencies of answers to each of the questions were determined and comparisons of categorical variables were made using a chi-square test or Fisher's exact test. Continuous variables were compared using the unpaired 2-tailed *t*-test or the Mann-Whitney *U *test. Data are expressed as Mean ± SD for those variables that were normally distributed, or medians and interquartile range (25^th^-75^th ^percentile) for those with a non-normal distribution. All statistical analysis was performed using SPSS software version 19.0 for Macintosh (SPSS Inc., Chicago, Illinois) and a two-tailed p-value of < 0.05 was considered statistically significant. All data analyses were performed following data collection in 2009.

## Results

### Demographic of study participants

A total of 25,000 physicians (10% of the AMA membership) were invited and 512 (2%) completed the survey during a four-week data collection period. The average survey participant was a white male physician based in a suburban setting who has been in private group practice between 11 and 20 years and who sees between 51 and 100 patients per week, Table 1. Notably, family practice physicians were more likely to be women compared to gastroenterologists (35 vs. 11%, *p *< 0.001). Non-white physicians were significantly more likely to be internists compared to their white peers (46.7 vs. 35.6%, *p *= 0.04).

**Table 1:** Baseline Demographic Characteristics of the survey respondents

Characteristics	Physicians, n (%)
Overall response n,(%)	512 (2)

Male, n (%)	376 (73.4)

Ethnicity	
White	390 (76.2)
Hispanic	8 (1.6)
African-American	9 (1.8)
Other	105 (20.5)

Specialty	
Family medicine	154 (30.1)
Internal medicine	196 (38.3)
Gastroenterology	145 (28.3)
Other	17 (3.3)

Practice location	
Urban, inner city	102 (19.9)
Urban, non-inner city	136 (26.6)
Suburban	200 (39.1)
Rural	74 (14.5)

Years in practice	
< 5	67 (13.1)
10-May	133 (26.0)
20-Nov	217 (42.4)
> 20	95 (18.6)

### Characteristics of the practice settings

While most study participants (18.4%) were from California, there were representatives from 28 states. A total of 40.8% of the respondents were from low CRC incidence states, 33.7% were from moderate, and 25.5% were from high CRC incidence states. Physicians in urban inner city settings were significantly more likely to be in practice for less than 5 years compared to their colleagues in suburban settings (39 vs. 14%, *p *< 0.001). Similarly, physicians in academic settings were more likely to be in practice for less than 5 years compared to their counterparts in solo private, solo group, or hospital-based practices (52%, 8%, 11%, and 36% respectively, *p *< 0.001). Family practice physicians were more likely to report practicing in a rural setting compared to gastroenterologists (22 vs. 10%, *p *= 0.013). The prevalence of solo private practitioners was highest among internists compared to gastroenterologists and family practice physicians (19.4%, 15.2%, and 8.4% respectively, *p *< 0.001).

### Knowledge of colorectal cancer screening guidelines

**Figure 1:**
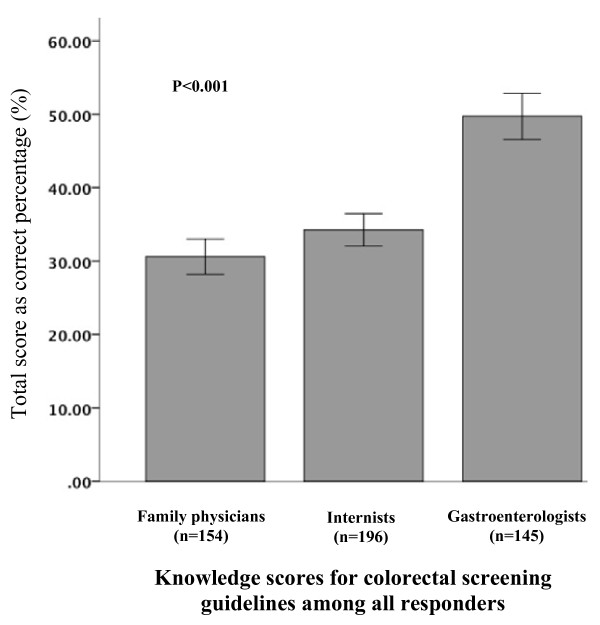
**Total knowledge score, as a percentage, for 154 family practice physicians, 196 internists, and 145 gastroenterologists**.

Among all responders, the average score on items assessing knowledge of CRC screening guidelines was only 37 ± 18%. Gastroenterologists averaged higher scores compared to internists and family physicians (50 ± 19%, 34 ± 15%, and 31 ± 15% respectively, *p *< 0.001), Figure 1. Knowledge scores were not significantly different when analyzed according to whether respondents were from low, moderate, or high CRC rate states (37 ± 19%, 37 ± 18%, and 39 ± 18% respectively, *p *= 0.30). Consistent with this finding scores did not differ significantly according to whether respondents characterized the practice location as urban inner city, urban non-inner city, suburban, or rural (35 ± 17%, 40 ± 20%, 37 ± 17%, and 39 ± 20% respectively, *p *= 0.26).

While 92% of physicians correctly identified age 50 as the point to initiate CRC screening for an average risk Asian patient, only 28% correctly identified age 45 as the ACG recommended initiation point for an African-American patient. Black physicians were the subgroup more likely to correctly identify the recommended CRC screening initiation age for African-American patients compared to their peers (66.7 vs. 27.8%, *p *= 0.01). For a high-risk patient whose grandmother had colorectal cancer at age 65, only 12% of physicians correctly identified the screening initiation point and follow-up interval.

### Physician identified barriers to CRC screening

Among all study participants 43% reported that there were barriers that influenced their ability to refer high-risk patients for colorectal cancer screening based on current established guidelines. The majority of the 219 physicians (72%) reporting barriers to CRC screening identified two or more factors. "Patient refusal of CRC screening" was cited by 69% of those reporting barriers, while 64% identified "lack of insurance reimbursement for early referral for colonoscopy" as a barrier. "Patient anxiety about testing" was a barrier for 58%, "unawareness of the current guidelines" for 23%, "lack of evidence to support efficacy of earlier screening" for 14%, while 12% cited "time constraints on taking a full family history" as a barrier. Physicians who reported that they were unaware of the CRC screening guidelines achieved significantly lower scores on the knowledge questions compared to their counterparts (31 ± 12 vs. 40 ± 18%, *p *= 0.002).

### Patterns in cited barriers to CRC screening

**Figure 2:**
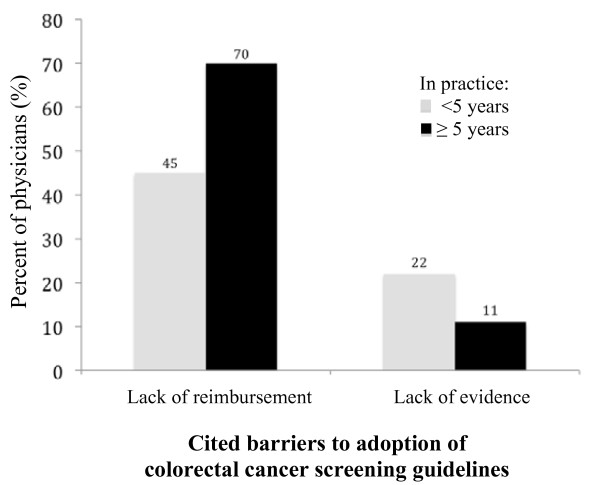
**Comparison of percentage of physicians reporting "lack of insurance reimbursement for early referral for colonoscopy" and "lack of evidence to support efficacy of earlier screening" as barriers to colorectal cancer screening guidelines, according to whether they have been in practice for < 5 or ≥ 5 years**.

The frequency of the cited barriers to referring high-risk patients for CRC screening did not differ according to physician practice location. Physicians in practice for less than 5 years were less likely to report "lack of insurance reimbursement" as a barrier to refer high-risk patients for CRC screening compared to their colleagues who have been in practice longer (45 vs. 70%, *p *< 0.001), but were more likely to cite "lack of evidence to support early screening" as a barrier (22 vs.11%, *p *= 0.04), Figure 2. Academic physicians were significantly less likely to cite "lack of insurance reimbursement" as a barrier to referral compared to physicians who described their practice as solo private, group private, and hospital-based (49%, 86%, 67%, and 70%, respectively, *p *= 0.005).

## Discussion

Our study assessed physician knowledge of nationally accepted CRC screening guidelines for high-risk individuals and the perceived barriers to their screening practices. Overall knowledge of CRC screening guidelines was low regardless of the CRC incidence rate in the state where the respondent practices. Among all of the practitioners, gastroenterologists scored the highest and were more likely to follow CRC screening guidelines than any other specialty. There was a notable lack of knowledge about differences in screening recommendations based on race/ethnicity.

Most physicians reported at least two barriers as adversely affecting their screening practices. The most frequently identified barrier to following CRC screening guidelines for high-risk patients was patient refusal, followed by a lack of insurance reimbursement and patient anxiety. The "patient refusal" barrier may be effectively addressed through targeted community outreach and education campaigns [19]. While Medicare provides coverage for CRC screening of high-risk patients without a minimum age, Medicaid coverage varies by state as do private insurance programs.

Not surprisingly, physicians who cited a lack of awareness of current guidelines also scored significantly lower on knowledge questions compared to their counterparts. In the future, this could be addressed by studying how physicians are staying informed about updated guidelines. Consistent with a previous report that used a "5-year" cut-off to stratify physicians [20] our study demonstrates that there is a significant relationship between a physician's mode of practice and the amount of years they have been in practice. It is notable that compared to their experienced counterparts physicians with less than 5 years practice experience were more likely to cite a "lack of evidence supporting the efficacy of earlier screening" using colonoscopy for high risk patients despite published evidence supporting structural evaluation of the colon [21].

Consistent with a previous regional study of physician knowledge and practice patterns [22], we observed differences in knowledge and screening strategy implementation between primary care physicians and gastroenterologists. As previously reported [22] and as noted in our study, physicians cited a lack of time to inquire about a family history as a barrier to screening. The importance of this barrier is underscored by the fact that appropriate implementation of CRC screening recommendations requires proper risk stratification of patients. As a consequence, individuals at high risk for CRC screening secondary to hereditary factors could be overlooked and may not be screened appropriately. Our findings are also in line with prior studies that have reported "patient refusal" and "patient anxiety about testing" as physician-identified barriers to CRC screening [10,11].

While this national survey of US physicians provides an important assessment of knowledge and practice patterns regarding CRC screening, a few limitations should be considered. First, the absolute number of physicians participating in the study was relatively small and white male physicians were the most likely respondents. However to our knowledge this is one of the largest national samples evaluating multiple physician specialties. In addition, the results of this study were based on physicians' self-reported practices from clinical vignettes and may not match actual practice. It should also be noted that while we referenced two established guidelines, variations exist in published guidelines and to date only the ACG recommends earlier screening for African Americans. Lastly, there was a higher representation from states with low CRC rates versus those with high rates, which may have had an unspecified influence in knowledge and practice patterns that could not be assessed by our study. Nevertheless the strengths of our study include physician representation from 28 states and fairly balanced response rates among providers in three specialties: internists, family practice physicians and gastroenterologists.

## Conclusions

In conclusion, this national survey reveals poor knowledge of CRC screening guidelines for high-risk populations across medical specialties. The opportunity exists to educate health care providers to use up-to-date screening recommendations. Besides lack of awareness of guidelines for high-risk patients, lack of reimbursement was cited as an adverse factor to CRC screening practices. Efforts to improve knowledge of screening guidelines among physicians and to standardize insurance coverage of CRC screening in high-risk patients may have the potential of improving existing disparities in colorectal cancer screening rates.

## Competing interests

The authors declare that they have no competing interests.

## Authors' contributions

PMW participated in statistical analysis, and manuscript preparation. MS participated in the design of the study, statistical analysis, and manuscript preparation. MAP participated in the design of the study, and manuscript preparation. FF participated in the design of the study, statistical analysis, and manuscript preparation. All authors read and approved the final manuscript.

## Supplementary Material

Additional file 1 **Appendix 1. Survey instrument sent to 25,000 physicians across the USA**. Click here for file

Additional file 2 **Appendix 2. Guidelines for Colorectal Cancer Screening: American College of Gastroenterology 2008**. Click here for file
