# Supplementary material for: Colorectal cancer screening of high-risk populations: A national survey of physicians
Source: BMC Res Notes. 2012 Jan 24;5:64. doi: 10.1186/1756-0500-5-64 (PMC3284403; doi:10.1186/1756-0500-5-64)
Supplement: Additional file 1 — Appendix 1. Survey instrument sent to 25,000 physicians across the USA. [file 1756-0500-5-64-S1.DOC]

**Appendix 1. Survey instrument sent to 25,000 physicians across the USA**

**Knowledge and perceived barriers questions**

1. A patient’s father had adenomatous polyps at age 55. At what age would you recommend screening this patient for colorectal cancer, and if the exam is normal how often would you screen?
2. Start age 40 and every 5 years.
3. Start age 40 and every 10 years.
4. Start age 45 and every 5 years.
5. Start age 45 and every 10 years.
6. Start age 50 and every 5 years.
7. Start age 50 and every 10 years.

(Answer: F)

1. A patient’s mother had colorectal cancer at age 70. At what age would you recommend screening this patient for colorectal cancer and if the exam is normal how often would you screen?
2. Start age 40 and every 5 years.
3. Start age 40 and every 10 years.
4. Start age 45 and every 5 years.
5. Start age 45 and every 10 years.
6. Start age 50 and every 5 years.
7. Start age 50 and every 10 years.

(Answer: F)

1. A patient’s father and brother had colorectal cancer diagnosed in their 70s. At what age would you recommend screening this patient and if the exam is normal how often would you screen?
2. Start age 40 and every 5 years.
3. Start age 40 and every 10 years.
4. Start age 45 and every 5 years.
5. Start age 45 and every 10 years.
6. Start age 50 and every 5 years.
7. Start age 50 and every 10 years.

(Answer: A)

1. A patient’s grandmother had colorectal cancer diagnosed at age 65. At what age would you recommend screening this patient and if the exam is normal how often would you screen?
2. Start age 40 and every 5 years.
3. Start age 40 and every 10 years.
4. Start age 45 and every 5 years.
5. Start age 45 and every 10 years.
6. Start age 50 and every 5 years.
7. Start age 50 and every 10 years.

(Answer: F)

1. At what age would you recommend starting to screen your Asian-American patients with no family history of colorectal cancer?
2. Start Age 40
3. Start Age 45
4. Start Age 50
5. Start Age 55

(Answer: C)

1. At what age would you recommend starting to screen your African-American patients with no family history of colorectal cancer?
2. Start Age 40
3. Start Age 45
4. Start Age 50
5. Start Age 55

(Answer: B)

1. A patient was told that he has a family history of Familial Adenomatous Polyposis (FAP) but has not been genetically tested. At what age would you recommend starting screening and how often?
2. Start sigmoidoscopy age 10 and then annually.
3. Start sigmoidoscopy age 20 and then annually.
4. Start sigmoidoscopy age 30 and then annually.
5. Start colonoscopy at age 50 and then every 10 years.

(Answer: B)

1. A patient was told he has a family history of Hereditary Nonpolyposis Colon Cancer (HNPCC). At what age would you recommend starting screening and how often?
2. Start age 20–25, and every 1–2 years or 10 years earlier than youngest CRC diagnoses.
3. Start age 10, and every 1–2 years or 10 years earlier than youngest CRC diagnoses.
4. Start age 50 and every 10 years

(Answer: A)

**Demographic questions**

1. Are there factors that influence your ability to refer high-risk patients for colonoscopy based on the current guidelines?
2. Yes
3. No
4. If yes to the above question: which of the following are barriers to you sending high-risk patients for earlier colonoscopies? (circle all the apply)
5. Time constraints on taking full family history
6. Unaware of the current guidelines
7. Lack of insurance reimbursement for early referral to colonoscopy
8. Patient anxiety about testing
9. Lack of evidence to support efficacy
10. Patient refusal
11. With what ethnic group would you identify yourself?
12. White
13. Hispanic
14. African-American
15. Asian-American
16. Other: please specify _______
17. What is your gender?
18. Male
19. Female
20. In which state is your primary practice located? _________
21. Which of the following best describes your primary-practice location?
22. Urban, inner-city
23. Urban, non-inner city
24. Suburban
25. Rural
26. How would you characterize your practice primarily?
27. Family Medicine
28. Internal Medicine
29. Gastroenterology
30. Surgery
31. Other ________
32. How many years have you been in practice?
33. <5
34. 5–10
35. 10–20
36. >20
37. How many patients do you see per week?
38. <25
39. 25–50
40. 50–100
41. >100
42. Do you refer patients for colonoscopy without prior GI consultation (open access colonoscopy)? Only answer if you are not a Gastroenterologist.
43. Yes
44. No
45. Which one of the following best describes your practice setting:
46. Academic
47. Private Practice - Solo
48. Private Practice - Group
49. Hospital Based Practice
