# Supplementary material for: Colorectal cancer screening of high-risk populations: A national survey of physicians
Source: BMC Res Notes. 2012 Jan 24;5:64. doi: 10.1186/1756-0500-5-64 (PMC3284403; doi:10.1186/1756-0500-5-64)
Supplement: Additional file 2 — Appendix 2. Guidelines for Colorectal Cancer Screening: American College of Gastroenterology 2008. [file 1756-0500-5-64-S2.DOC]

**Appendix 2. Guidelines for Colorectal Cancer Screening: American College of Gastroenterology 200818**

|  | |
| --- | --- |
| **Preferred CRC screening recommendations**   - - Cancer prevention tests should be offered first. The preferred CRC prevention test is colonoscopy every 10 years, beginning at age 50. Screening should begin at age 45 years in African Americans   - Cancer detection test. This test should be offered to patients who decline colonoscopy or another cancer prevention test. The preferred cancer detection test is annual Fecal Immunochemical Test for blood   **Alternative CRC prevention tests**   - - Flexible sigmoidoscopy every 5–10 years   - CT colonography every 5 years   **Alternative cancer detection tests**   - Annual Hemoccult Sensa - Fecal DNA testing every 3 years | **Familial Adenomatous Polyposis**   - Patients with classic FAP (>100 adenomas) should be advised to pursue genetic counseling and genetic testing, if they have siblings or children who could potentially benefit from this testing - Patients with known FAP or who are at risk of FAP based on - family history (and genetic testing has not been performed) - should undergo annual flexible sigmoidoscopy or colonoscopy, as appropriate, until such time as colectomy is deemed by physician and patient as the best treatment - Patients with retained rectum after subtotal colectomy should undergo flexible sigmoidoscopy every 6 – 12 months - Patients with classic FAP, in whom genetic testing is negative, should - undergo genetic testing for bi-allelic MYH mutations. Patients with - 10 – 100 adenomas can be considered for genetic testing for attenuated FAP and if negative, MYH associated polyposis |
| **Recommendations for screening when family history is positive but evaluation for HNPCC considered not indicated**   - Single first-degree relative with CRC or advanced adenoma diagnosed at age ≥ 60 years. Recommended screening: same as average risk - • Single first-degree with CRC or advanced adenoma diagnosed at age < 60 years or two first-degree relatives with CRC or advanced adenomas. Recommended screening: colonoscopy every 5 years beginning at age 40 years or 10 years younger than age at diagnosis of the youngest affected relative | **Hereditary Non-polyposis Colorectal Cancer**   - Patients who meet the Bethesda criteria should undergo microsatellite instability testing of their tumor or a family member’s - tumor and/or tumor immunohistochemical staining for mismatch - repair proteins - Patients with positive tests can be offered genetic testing. Those - with positive genetic testing, or those at risk when genetic testing - is unsuccessful in an affected proband, should undergo colonoscopy every 2 years beginning at age 20 – 25 years, until age 40 years, then annually thereafter |
|  |  |

*CRC* colorectal cancer; *CT* computed tomography, *FAP* familial adenomatous polyposis; *FIT* fecal immunochemical test; *HNPCC* hereditary non-polyposis colorectal cancer
